# In Search of Small Molecule Inhibitors Targeting the Flexible CK2 Subunit Interface

**DOI:** 10.3390/ph10010016

**Published:** 2017-02-03

**Authors:** Benoît Bestgen, Zakia Belaid-Choucair, Thierry Lomberget, Marc Le Borgne, Odile Filhol, Claude Cochet

**Affiliations:** 1 Biology of Cancer and Infection, INSERM, U 1036, 38054 Grenoble, France; benoit.bestgen@gmail.com (B.B.); odile.filhol-cochet@cea.fr (O.F.); 2 Biology of Cancer and Infection, University Grenoble-Alpes (UGA), 38000 Grenoble, France; 3 Commissariat à l’Energie Atomique et aux Energies Alternatives (CEA), Direction de Recherche Fondamentale (DRF), Biosciences and Biotechnology Institute of Grenoble (BIG), Biology of Cancer and Infection (BCI), 38054 Grenoble, France; 4 Pharmaceutical and Medicinal Chemistry, Saarland University, Campus C2.3, 66123 Saarbrücken, Germany; 5 Faculté de Pharmacie—ISPB, EA 4446 Bioactive Molecules and Medicinal Chemistry, SFR Santé Lyon-Est CNRS UMS3453—INSERM US7 Université Lyon 1, 8 avenue Rockefeller, F-69373 Lyon CEDEX 8, France; thierry.lomberget@univ-lyon1.fr (T.L.); marc.le-borgne@univ-lyon1.fr (M.L.B.); 6 INSERM U 1163 / CNRS ERL 8254, Institut IMAGINE, Université Paris Descartes, 75015 Paris, France; zbelaidchoucair@gmail.com

**Keywords:** protein kinase CK2, subunit interface, cyclic peptides, protein–protein interaction, cell death

## Abstract

Protein kinase CK2 is a tetrameric holoenzyme composed of two catalytic (α and/or α’) subunits and two regulatory (β) subunits. Crystallographic data paired with fluorescence imaging techniques have suggested that the formation of the CK2 holoenzyme complex within cells is a dynamic process. Although the monomeric CK2α subunit is endowed with a constitutive catalytic activity, many of the plethora of CK2 substrates are exclusively phosphorylated by the CK2 holoenzyme. This means that the spatial and high affinity interaction between CK2α and CK2β subunits is critically important and that its disruption may provide a powerful and selective way to block the phosphorylation of substrates requiring the presence of CK2β. In search of compounds inhibiting this critical protein–protein interaction, we previously designed an active cyclic peptide (Pc) derived from the CK2β carboxy-terminal domain that can efficiently antagonize the CK2 subunit interaction. To understand the functional significance of this interaction, we generated cell-permeable versions of Pc, exploring its molecular mechanisms of action and the perturbations of the signaling pathways that it induces in intact cells. The identification of small molecules inhibitors of this critical interaction may represent the first-choice approach to manipulate CK2 in an unconventional way.

## 1. Introduction

**Figure 1:**
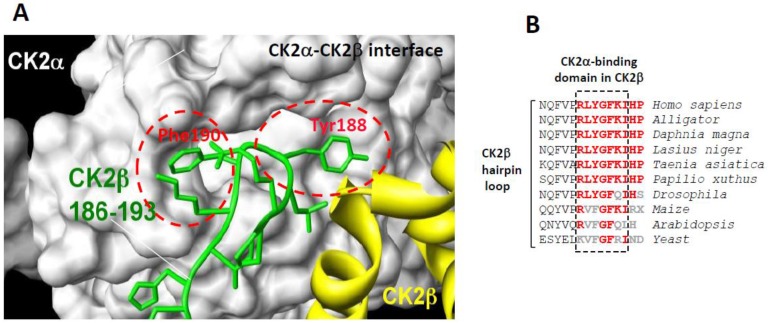
(**A**) surface representation of the binding pocket of CK2α interacting with a short C-terminal hairpin loop of CK2β adapted from [15]. Crystal structure of the CK2 holoenzyme shows that a C-terminal fragment of the CK2β1chain encompassing residues 186–193 (green) forms a loop inserting into a deep hydrophobic pocket of CK2α [11]. Surface representation of the CK2α cleft in grey highlights its pocket-like characteristics. The phenol and phenyl rings of the two non-polar and aromatic CK2β residues Tyr188 and Phe190, respectively, (red) are in quasi-planar opposite orientation and fit tightly into it; and (**B**) a highly conserved cluster in the C-terminal domain of CK2β in different species.

Protein Kinase CK2 exhibits a heterotetrameric quaternary structure composed of two catalytic (α and/or α’) subunits linked to a stable dimer of two regulatory (β) subunits. In contrast to other multi-subunit protein kinases, the free catalytic α/α’ subunits are constitutively active, and the regulatory β subunits act as targeting subunits controlling the substrate specifity and cellular localization of the holoenzyme complex. As a pro-survival kinase, CK2 has emerged as a relevant therapeutic target being dysregulated in various cancers and several human pathologies, therefore supporting the development of chemical inhibitors as promising drug candidates [1,2,3,4]. Most CK2 inhibitors identified during the last two decades are ATP-competitive molecules [5,6] with one of them, CX-4945, being investigated in phase I and II clinical trials [7,8,9]. CK2 is endowed with a peculiar molecular architecture suggesting the existence of different exosites distinct from the catalytic cavity that can be targeted by small molecules to achieve functional effects [10]. The inter-subunit flexibility suggested by X-ray crystallography studies [11] and live-cell fluorescent imaging [12], together with the imbalance in the expression of CK2 subunits in various tumors [13], suggest that their interaction is a key point of regulation. This offers attractive opportunities for the identification of small molecules that modulate this interaction. Therefore, we focused our attention on the CK2α/CK2β interface [11,12], which is relatively small (832 Å^2^), and harboring a suitable binding pocket for small molecules [14]. In the CK2 holoenzyme structure, a segment located in the CK2β tail points away from the protein core and forms a β-hairpin loop that inserts deep into a shallow hydrophobic groove present in the β4/β5 sheets of CK2α [11] (Figure 1A). Notably, the sequence of this C-terminal hairpin loop of CK2β is highly conserved in different species (Figure 1B). Site-directed mutagenesis and functional assays have revealed that only a small set of primary hydrophobic residues (Tyr188 and Phe190) present in this CK2β hairpin loop dominates affinity [15]. Further characterization of these hotspots led to the structure-based design of CK2β-derived peptides that were cyclized through two cysteines forming one disulfide bridge. One of these conformationally constrained cyclic peptides, initially termed Pc, was able to efficiently antagonize the assembly of the CK2 holoenzyme complex and to strongly affect its substrate preference [15], indicating that this peptide adopts an energetically favorable state. This 13-mer peptide (GCRLYGFKIHGCG) was the first small antagonist that binds to the CK2 interface and inhibits its high affinity subunit interaction. Based on CK2β sequence, alanine scanning mutagenesis of Pc revealed a cluster of residues (Tyr188 and Phe190) essential for its bioactivity [15]. Moreover, a structural rationalization of the CK2β-competitive potential of Pc was provided by the X-ray structure of a Pc-CK2α complex [16]. Recently, comparative molecular dynamics simulations performed on this complex highlighted, among the hydrophobic residues, the prominent role of Phe190 for a stable and active conformation of Pc [17]. Thus, as a high affinity subunit interaction inhibitor, Pc is a promising CK2 antagonist candidate. However, structural modifications of this cyclic peptide, such as attachment to cell-permeant adducts, head-to-tail cyclization or biotin conjugation, are respectively required to confer in vivo activity or for its detection in cell extracts. We performed these studies with the aim to characterize the molecular mechanisms of action of Pc in intact cells and to investigate how this different mode of CK2 inhibition, through the perturbation of signaling pathways, will translate into phenotypes.

## 2. Results and Discussion

### 2.1. Structure and Mode of Binding of CK2β on CK2α

The high-resolution structure of the CK2 holoenzyme has revealed that the subunit interface is composed of: (1) hydrophobic residues in a groove located N-terminally at the outer surface formed by the juxtaposition of the antiparallel β4/β5 sheets of CK2α and (2) a cluster of well-defined hydrophobic residues present on the CK2β chain. This cluster, which contains highly conserved residues (R_186_LYGFKIH_193_) in different species (Figure 1B), exhibits a specific structural feature: it points away from the protein core and forms a 90° β-hairpin loop with Tyr188 at its top, which binds into a shallow hydrophobic groove present in the β4/β5 sheets of CK2α [11]. The interface relies on the steric complementarity between this CK2α groove and the hydrophobic face of the CK2β hairpin loop and, in particular, on a triad of CK2β amino acids, Tyr188, Gly189 and Phe190, which inserts deep into the CK2α groove (Figure 1A). The existence of this potential druggable pocket within the interface area of CK2 was corroborated by a structural study showing that a CK2 inhibitor, 5,6-dichloro-1-β-d-ribofuranosylbenzimidazole (DRB), binds, in addition to the canonical ATP cleft, to the CK2α/CK2β interface [14].

### 2.2. Rational Design of CK2β-Derived Cyclic Peptides

**Figure 2:**
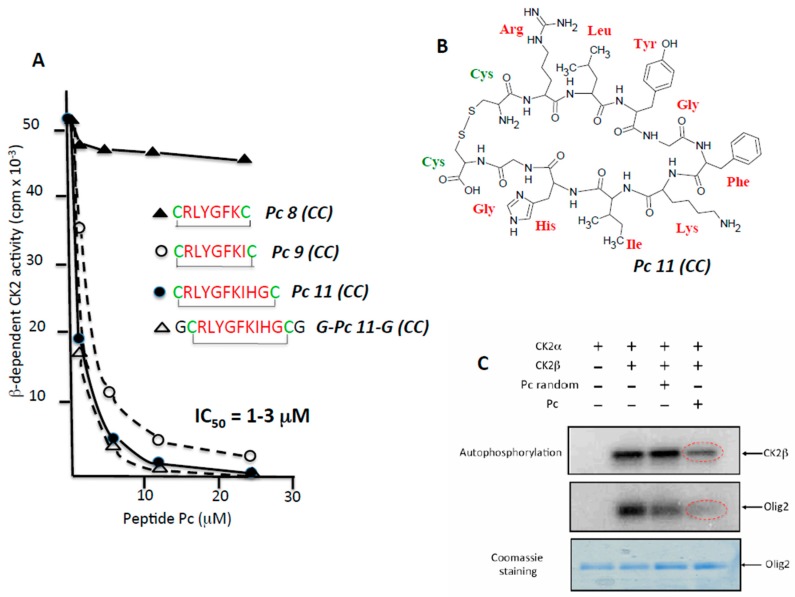
CK2β-derived cyclic peptides inhibit CK2 activity in vitro. (**A**) a series of cyclized CK2β-derived peptide analogues varying in length from eight to 11 amino acids were tested for their effect on the phosphorylation of eIF2β-derived peptide substrate whose phosphorylation relies on the holoenzyme formation. Representative data shown from two independent experiments; (**B**) structure of Pc 11 (CC); (**C**) antagonist effect of Pc 11 (CC) on CK2-mediated phosphorylation of the CK2β-dependent substrate Olig2. CK2α was incubated alone or with CK2β in the absence or presence of 25 µM random Pc 11 (CC) or Pc 11 (CC) for 15 min at 4 °C, followed by the addition of GST (Gluthatione *S*-Transferase)-Olig2 (5 μg) and 25 μM [γ-^32^P]ATP/MgCl_2_ for 5 min at room temperature. Phosphorylated proteins were separated by SDS/PAGE (Sodium dodecylsulfate/ Polyacrylamide gel electrophoresis) and analyzed by autoradiography. The gel was stained with coomassie blue to visualize GST-Olig2.

In a previous study, we used the X-ray structure of CK2β in the holoenzyme complex as a template for the design of a conformationally constrained 13-mer peptide (Pc 13) derived from the CK2β C-terminal domain and centred around the Tyr188 and Phe190 hotspots. Pc 13 is an eight-residue peptide (Arg186–His193) that contains a cluster of hydrophobic residues (Leu187, Tyr188 and Ile192) and three additional glycine residues, cyclized via two cysteine residues to ‘staple’ its conformation and to mimic the binding face of CK2β with CK2α [15]. A comparative analysis of the effect of varying the length from 11 to eight amino acids in a series of cyclized peptide analogues was performed by testing their effect on the phosphorylation of a peptide substrate (eIF2β peptide) whose phosphorylation relies on the holoenzyme formation [18]. Although most length variants inhibited the CK2β-dependent phosphorylation of this peptide to the same extent (IC_50_ = 1–3 µM), the 8-residue peptide (Pc 8 (CC)) was inactive (Figure 2A). Noteworthy, a linear form of Pc 11 (CC) or an inverted sequence were without effect (not shown), indicating that both the sequence and the constrained conformation of the peptide are essential for its antagonist activity. The cyclized structure of Pc 11 (CC) peptide that was used in the following experiments is shown in Figure 2B. A functional assay of the Pc 11 (CC) peptide was also performed testing its effect on the phosphorylation of the Olig-2 transcription factor, which relies exclusively on the tetrameric form of CK2 [19]. Figure 2C shows that the presence of Pc 11 (CC) led to a strong decrease of the original Olig-2 phosphorylation. Similarly, the phosphorylation of CK2β in the holoenzyme, an indicator of CK2 oligomerization [20], was significantly affected by the presence of Pc 11 (CC), reflecting a Pc-induced dissociation of the catalytically active CK2 holoenzyme complex.

### 2.3. Pc Binds to CK2β in CK2β-Deficient Cell Extracts

**Figure 3:**
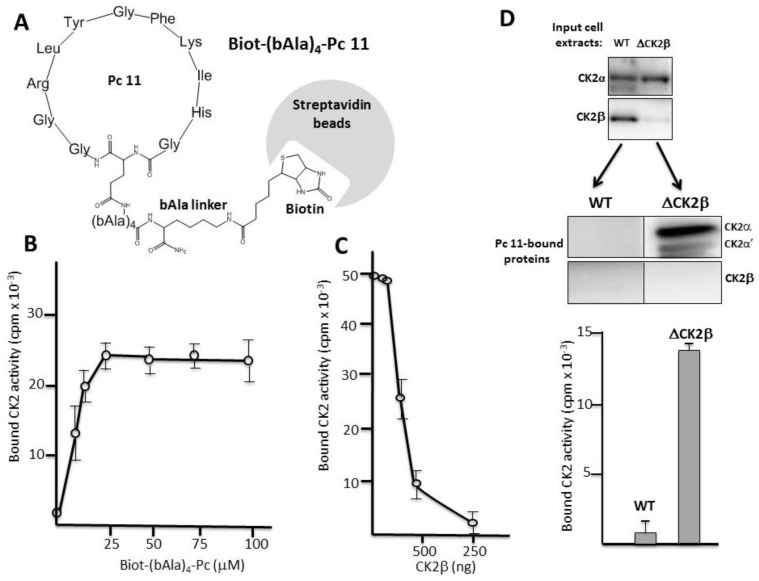
Pc binds to CK2α. (**A**) Biot-(bAla)_4_-Pc: Head-to-tail cyclic Pc 11 analogue linked to biotin through a 4-Ala linker; (**B**) aliquots of streptavidin-agarose beads were incubated with increasing concentrations of Biot-(bAla)_4_-Pc 11 in the presence of CK2α (100 ng) for 2 h at 4 °C. After three washes, CK2 activity assays were carried out for 5 min on the beads pellets. Representative data shown from two independent experiments. Results are presented as mean ± s.d. of triplicates; (**C**) aliquots of streptavidin-agarose beads were loaded with 25 µM Biot-(bAla)_4_-Pc 11 and incubated with CK2α (100 ng) in the presence of increasing amounts of CK2β for 2 h at 4 °C. After three washes, CK2 activity assays were carried out for 10 min on the beads pellets; (**D**) Pc binds to CK2α in cell extracts. Aliquots of streptavidin-agarose beads loaded with 25 µM Biot-(bAla)_4_-Pc 11 were incubated with WT or CK2β-depleted MCF-10A cell extracts (upper panel) for 2 h at 4 °C. After three washes, CK2 subunits retained on the beads were determined by Western blot (middle panel). CK2 activity was also determined in the bead pellets with the CK2β-independent peptide substrate (lower panel).

We designed a covalent head-to-tail cyclic Pc 11 analogue linked to biotin through a 4-bAla linker to circumvent any sterical hindrance. This Biot-(bAla)_4_-Pc 11 analogue was suitable for a pull-down assay using streptavidin-coated beads (Figure 3A). Recombinant CK2α was incubated with increasing concentrations of bead-bound Biot-(bAla)_4_-Pc 11 and after several washes, CK2 activity associated with the beads was determined. Figure 3B shows that, in this pull-down assay, the immobilized Pc peptide was efficient for a productive high-affinity interaction with CK2α. This interaction was strongly impaired by the presence of increasing concentrations of CK2β, showing that Pc 11 binds tightly to CK2α and behaves as an antagonist of the CK2 subunits interaction (Figure 3C). We then incubated Biot-(bAla)_4_-Pc 11-coated beads with WT (Wild Type) or CK2β-depleted MCF-10A cell extracts (Figure 3D upper panel). Western blot analysis showed that the free CK2α/α’ subunits present in CK2β-depleted MCF-10A cell extracts were recovered associated with the beads indicating a tight binding. In contrast, CK2 was not detected on beads incubated with WT cell extracts in which CK2β behaves as a Pc antagonist (Figure 3D lower panel).

### 2.4. Design and Characterization of a TAT-Conjugated Pc Analogue

**Figure 4:**
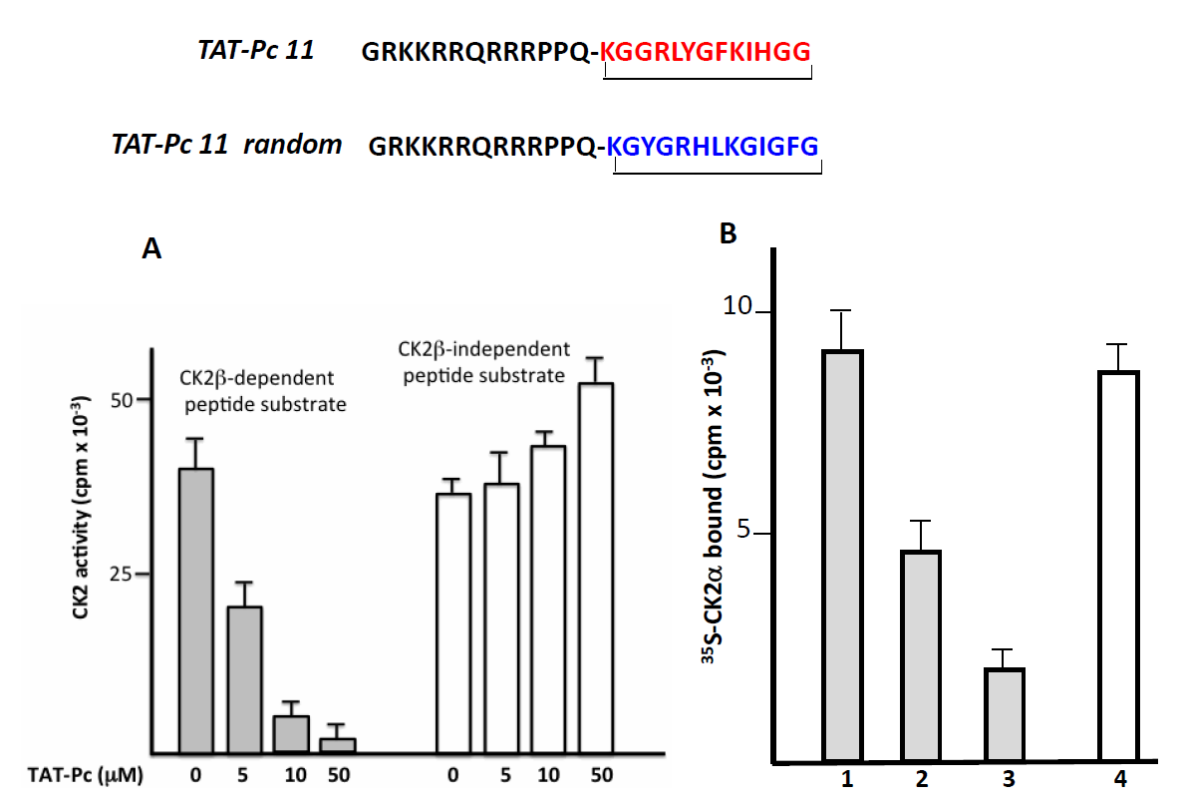
Inhibitory activity of a TAT-Pc analogue in vitro. (**A**) CK2 holoenzyme (100 nM) was incubated in the presence of increasing concentrations of TAT-Pc 13 for 15 min at 4 °C. CK2 activity was then determined with CK2β-dependent or independent peptide substrates; (**B**) [^35^S]-labeled CK2α was incubated with biotinylated MBP-CK2β immobilized on streptavidin coated plates (preformed complex) in the absence (1) or presence (2) of TAT-Pc 13. Alternatively, [^35^S]-labeled CK2α was incubated with TAT-Pc 13 (3) or random TAT-Pc 13 (4) and then with immobilized biotinylated MBP-CK2β. After washing, the amount of [^35^S]-labeled CK2α remaining in the complex was determined by radioactivity counting. Representative data shown from two independent experiments. Results are presented as mean ± s.d. of triplicates.

The successful transport of peptides/proteins to intracellular targets was accomplished by the use of a sequence derived from the HIV transactivator of transcription protein (TAT), which, when fused to cargo, facilitates receptor- and energy-independent transport across cell membranes [21]. As disulfide bonds are very sensitive to intracellular reductive conditions, head-to-tail cyclization is intuitively expected to improve bioactivities by increasing stability and lowering flexibility as well as sensitivity to proteolytic attack. Therefore, we fused the TAT sequence to the N-terminus of a covalent head-to-tail cyclized Pc 13 analogue (TAT-Pc 13, Figure 4). The biological properties of this peptide were first evaluated in vitro in a kinase assay using two different CK2 peptide substrates. As shown in Figure 4A, the CK2β-dependent phosphorylation of the eIF2β-derived peptide was strongly inhibited by increasing concentrations of TAT-Pc 13 (IC_50_ = 5 µM), whereas, as previously described [16], the CK2β-independent peptide phosphorylation was significantly stimulated at high TAT-Pc 13 concentrations. The bioactivity of TAT-Pc 13 was also tested in a CK2 subunit interaction assay. At a 50 µM concentration of TAT-Pc 13, the subunit interaction was inhibited by 80%. Interestingly, the addition of TAT-Pc 13 to a pre-formed α/β complex led to a significant reduction of CK2α binding (by more than 40%), suggesting a partial dissociation of the complex. In this assay, a TAT-conjugated random Pc 13 was without effect (Figure 4B). Thus, the TAT-conjugated Pc peptide retains inhibitory binding activity in vitro.

### 2.5. Bioactivity of TAT-Pc 13 in Cell Extracts

**Figure 5:**
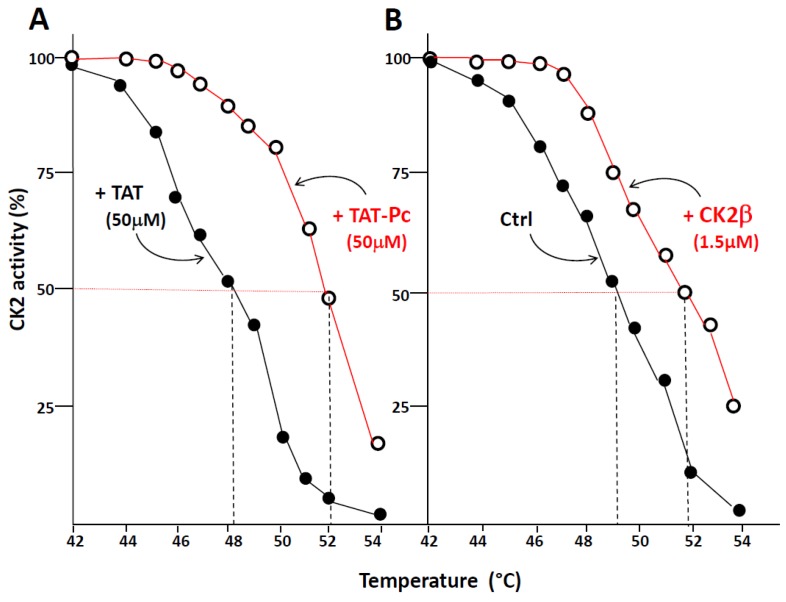
Thermal shift assay in cell lysates. CK2β-depleted MCF-10A cell lysates were treated with 50 µM TAT or TAT-Pc 13 (**A**), or with PBS (Phosphate-buffered saline) or CK2β (**B**) for 30 min at room temperature. The respective lysates were divided into 20 µL aliquots and heated individually at different temperatures for 3 min. After centrifugation, the supernatants were assayed for CK2 activity with the CK2β-independent peptide substrate. Representative data shown from two independent experiments in duplicate.

Thermal shift assay (TSA) is based on the biophysical principle of ligand-induced thermal stabilization of target proteins. This assay can be used to detect protein–ligand interactions in complex cell extracts. Therefore, we have generated thermal melting curves from cell extracts, in which the extent of CK2 unfolding was measured. The assays were performed on WT or CK2β-depleted MCF-10A cell extracts incubated with 50 µM of TAT or TAT-Pc 13 and exposed to increasing temperatures. Figure 5A shows that an obvious shift (4 °C) in the melting curves was detected in CK2β-depleted cell extracts incubated with TAT-Pc 13. Similarly, a significant shift (3 °C) was observed in cell extracts incubated with recombinant CK2β as compared to controls (Figure 5B). Of note, in extracts from WT MCF-10A cells expressing stoechiometric amount of CK2β, no thermal shift was observed upon incubation with TAT-Pc 13 (not shown). Thus, the ligand-induced stabilization of CK2α shows its engagement as a target for TAT-Pc 13 or CK2β in CK2β-depleted cell extracts.

**Figure 6:**
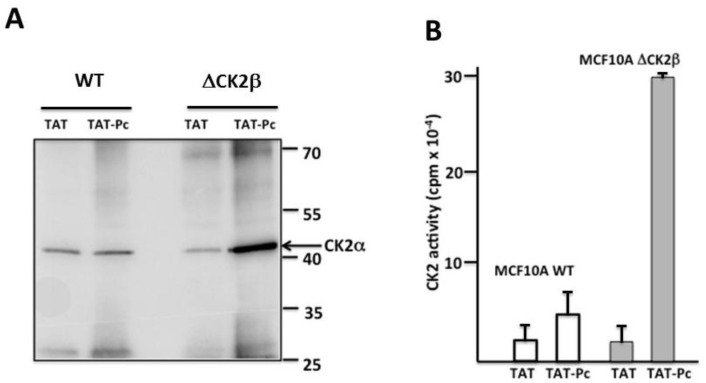
Physical binding of CK2α to TAT-Pc 13 in cell extracts. WT or CK2β-depleted MCF-10A cell lysates were incubated with 50 µM TAT or TAT-Pc 13 for 30 min at room temperature and immunoprecipitated with a TAT antibody. CK2α protein and CK2 activity were evaluated by Western blot (**A**) and CK2 kinase assay (**B**), respectively. Representative data for CK2 activity shown from two independent experiments. Results are presented as mean ± s.d. of triplicates.

In a second set of experiments, WT or CK2β-depleted MCF-10A cell extracts incubated with 50 µM of TAT or TAT-Pc 13 were immunoprecipitated with a TAT antibody and the presence of CK2α in the immunoprecipitates was evaluated by both Western blotting and CK2 kinase assays. In CK2β-depleted MCF-10A cell extracts, a higher amount of CK2α protein (Figure 6A) or CK2 activity (Figure 6B) were found associated with immunoprecipitated TAT-Pc 13 compared to TAT immunoprecipitates. In WT MCF-10A cell extracts, CK2α present in TAT-Pc 13 immunoprecipitates was not significantly different compared to TAT immunoprecipitates.

Altogether, these experiments show that TAT-Pc 13 can form a stable complex with CK2α in CK2β-depleted MCF-10A cell extracts, whereas, in WT cell extracts, this interaction was hampered by the presence of endogenous CK2β.

### 2.6. Uptake and Cellular Effects of TAT-Pc

**Figure 7:**
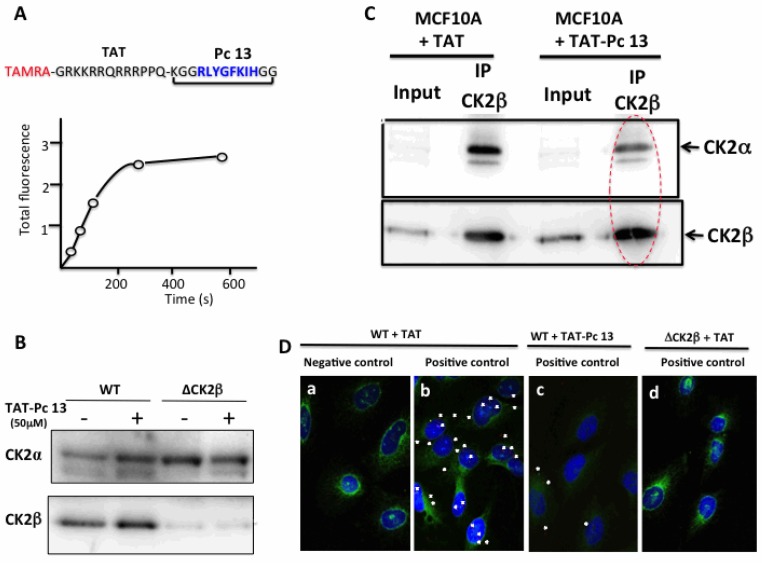
Uptake and cellular effects of TAT-Pc 13. (**A**) kinetic of TAMRA-TAT-Pc 13 uptake in MCF-10A cells; (**B**) WT or CK2β-depleted MCF-10A cells were incubated with 50 µM TAT or TAT-Pc 13 for 8 h and the expression of CK2 subunits was evaluated by Western blot; (**C**) anti-CK2β immunoprecipitates were prepared from WT MCF-10A cells incubated with 50 µM TAT or TAT-Pc 13 for 2 h. Presence of CK2 subunits in the corresponding immunoprecipitates was determined by Western blot; (**D**) in situ proximity ligation images of MCF-10A cells incubated with 50 µM TAT (a,b,d) or TAT-Pc 13 (c) for 6 h. WT cells were incubated with CK2α antibody alone (a) or CK2α and CK2β antibodies (b,c). CK2β-depleted cells (ΔCK2β) were incubated with 50 µM TAT and then with CK2α and CK2β antibodies (d). Actin staining in green, scale bar 10 µm.

To assess the cell-penetrating properties of TAT-Pc 13, we used a tetramethylrhodamine-conjugated TAT-Pc 13 analogue (TAMRA-TAT-Pc 13). The cellular uptake of this fluorescently labeled peptide was very rapid: only 2 min after incubation, the peptide was detectable in MCF-10A cells and plateauing after 5 min (Figure 7A). The impact of TAT-Pc 13 cell uptake on the expression of the CK2 subunits in WT and CK2β-depleted MCF-10A cells was evaluated by Western blot. This analysis showed that, after an 8 h-treatment with TAT-Pc 13, the level of CK2 subunits was not significantly changed in both cell types (Figure 7B). We next evaluated the effect of TAT-Pc 13 on the CK2 subunit association using two independent strategies. First, CK2β was immunoprecipitated in extracts of MCF-10A cells treated with TAT or TAT-Pc 13 and the amount of CK2α recovered in the immunoprecipitates was analyzed by Western blot. Figure 7C shows that CK2β immunoprecipitates from cells incubated with TAT-Pc 13 contained significantly less CK2α than cells treated with TAT, thus demonstrating that TAT-Pc 13 impaired CK2α/CK2β interaction in living cells.

To confirm these results, the effect of TAT-Pc was evaluated in MCF-10A cells, using the in situ proximity ligation assay (PLA). PLA is an antibody-based method representing a reliable readout of molecular proximity of two antigens located on two distinct proteins. In this method, two different proteins of interest are recognized by their respective specific primary antibody and then with a corresponding pair of secondary antibodies conjugated to complementary oligonucleotides. In close proximity (<40 nm), the oligonucleotides hybridize and are ligated and amplified. The fluorescent signal from each pair of PLA probes confirms a close proximity and not simply subcellular colocalization and can then be detected and quantified as fluorescent spots in microscopic images of the cells [22]. Our results illustrated in Figure 7D indicate a clear proximity between CK2α and CK2β subunits, attested by several fluorescent interaction dots in cells incubated with antibodies against both proteins (panel b, positive control, 4.8 ± 1.5 dots/cell), but not in a negative control sample (panel a) in which the CK2β primary antibody has been omitted. The specificity of the signal was also confirmed by turning down the expression of CK2β through RNA interference (see Figure 3D, upper panel). No signal was detected in CK2β-depleted cells (panel d). Remarkably, incubation of the cells with TAT-Pc 13 for 6 h strongly reduced the number of interaction dots (panel c, 2.2 ± 1.1 dots/cell). Collectively, these results show that TAT-Pc 13 has suitable properties to gain intracellular access and to disrupt the dynamic CK2 subunit interaction in living cells.

### 2.7. Inhibition of Cell Viability by TAT-Pc 13

**Figure 8:**
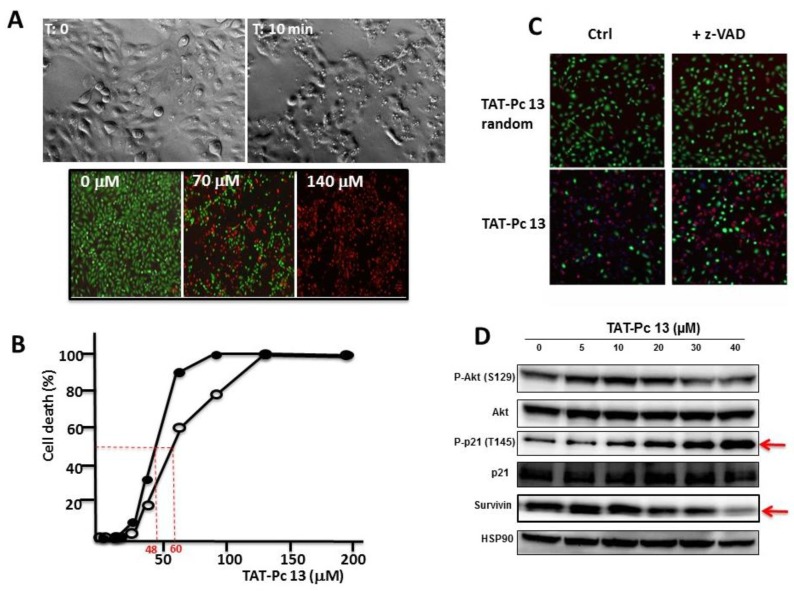
Cellular effects of TAT-Pc 13. (**A**) MCF-10A cells were incubated with 25 µM of TAT-Pc 13 for 3 h and imaged with phase microscopy (upper panel) or with increasing concentrations of TAT-Pc 13 for 2 h and cell viability was evaluated using the LIVE&DEAD™ assay. Live cells fluoresce bright green (530 nm), whereas dead cells fluoresce red-orange (645 nm) (lower panel, scale bare: 100 µm); (**B**) WT (○-○) or CK2β-depleted (●-●) MCF-10A cells were incubated for 4 h with increasing concentrations of TAT-Pc 13 and cell death was evaluated using the LIVE&DEAD™ assay recording fluorescence at 645 nm. Representative data shown from two independent experiments in duplicate; (**C**) MCF-10A cells were pre-treated in the absence or presence of 20 µM z-VAD for 5 h and then incubated for 4 h with 25 µM random TAT-Pc 13 or TAT-Pc 13 and cell viability was evaluated as in (A) (scale bare: 100 µm); and (**D**) intracellular events induced by increasing concentrations of TAT-Pc 13. After 12 h of treatment, 786-O cells were lysed and analyzed by Western blot.

TAT-Pc 13 was found to be an unusually rapid inducer of cell death. Three hours after TAT-Pc 13 was introduced into the cultures, more than 50% of the cells rounded up and shrank, suggesting cell death (Figure 8A upper panel). This was confirmed using the LIVE&DEAD^TM^ cell viability assay that provides a visual readout of cell integrity (Figure 8A lower panel). This cellular effect was also analyzed after treatment of WT or CK2β-depleted MCF-10A cells with increasing concentrations of TAT-Pc 13. TAT-Pc 13 caused a cell death in both cell types, which was already detectable at a concentration of 25 µM and became massive at 50–60 µM (Figure 8B). Compared to WT cells, CK2β-depleted MCF-10A cells were more sensitive to cell death (IC_50_ = 60 µM and 48 µM, respectively) suggesting that TAT-Pc 13-induced cell death is partially antagonized by CK2β present in WT cells. Notably, this cell death was insensitive to the pan-caspase inhibitor, z-VAD-FMK, indicating that the cytotoxic effect of TAT-Pc 13 relies on the induction of a caspase-independent cell death irrelevant to cell apoptosis (Figure 8C). Moreover, TAT-Pc 13 was found to induce cell death of various cancerous cell lines (HeLa, MDA MB-231, 786-0) indicating activity across a wide variety of cell lines (Supplementary Materials Figure S1). In order to investigate the intracellular events induced by TAT-Pc 13, MCF-10A cells were incubated with increasing concentrations of TAT-Pc 13 and analyzed by Western blot. Figure 8D shows no sign of AKT activation, whereas TAT-Pc 13 induced the phosphorylation of the tumor suppressor p21 on Thr145 and a decrease of survivin expression in a dose-dependent manner (EC_50_ = 15 µM). In contrast, phosphorylation of p21 was strongly inhibited in presence of CX-4945 (Supplementary Materials Figure S1).

## 3. Materials and Methods

### 3.1. Materials

The antibodies used for Western blotting were: rabbit anti-Akt and rabbit anti-anti-HSP90 (Cell Signaling Technology, Danvers, MA 01923, USA), rabbit anti-phospho-Akt (Ser129) from Abgent (San Diego, CA 92121, USA), rabbit anti-p21 and mouse anti-TAT were from Santa Cruz Biotechnologies (Dallas, Texas 75220, USA), rabbit anti-phospho-p21 (Thr145) from Abcam (Paris, 75010, France), and rabbit anti-Survivin (Novus Biologicals, Littleton, CO 80120, USA). Mouse anti-CK2β and rabbit anti-CK2α were previously described [13,23].

HRP (Horse Raddish Peroxidase)-conjugated secondary goat anti-rabbit IgG antibodies were from Jackson Immunoresearch (West Grove, PA 19390, USA). Wild type (WT) MCF-10A cells were obtained from ATCC (Middlesex, TW11, UK). Stable CK2β silencing in MCF-10A cells (ΔCK2β) was accomplished by transduction with pLKO1 lentivirus (Sigma-Aldrich, St. Louis, MO 63178, USA), followed by puromycin selection as previously described [13]. The cells tested negative from mycoplasma contamination were grown as described [24].

### 3.2. Peptide Synthesis

Cystein-bridged cyclic peptides and biotinylated cyclic peptides were obtained from Genosphere Biotechnologies (Paris, France). TAT-Pc 13 was obtained from GeneCust (Dudelange, Luxembourg). TAMRA-TAT-Pc 13 was synthesized by VCPBIO (Shenzhen, China).

### 3.3. Proteins

Production and purification of recombinant CK2α and CK2β proteins were described previously [25,26,27].

### 3.4. In Vitro Kinase Assays

CK2 kinase assays were performed in a phosphorylation buffer containing recombinant CK2α (36 ng) or recombinant CK2 holoenzyme (50 ng) and a mixture containing 10 mM MgCl_2_ and 1 μCi of [γ-32P]ATP and 0.15 M NaCl in the presence of 0.2 mM of the CK2β-independent substrate RRREDEESDDEE [28] or the CK2β-dependent substrate MSGDEMIFDPTMSKKKKKKKKP [18]. The final concentration of ATP was 100 μM and assays were performed under linear kinetic conditions for 5–10 min at room temperature. Kinase reactions were terminated by the addition of 60 μL of 4% trichloroacetic acid and sample adsorption on phosphocellulose P81 paper, which were washed in 0.5% phosphoric acid and counted in a scintillation counter.

### 3.5. In Vitro Pull-Down Assay

Streptavidin-agarose beads (Fluka #85881) were equilibrated in binding buffer A (50 mM Tris-HCl pH 7.5, 0.2 M NaCl, 0.1% Tween 20) containing 1 mg/mL BSA. After several washes in buffer B (50 mM Tris-HCl pH 7.5, 0.4 M NaCl, 0.1% Tween 20), beads were incubated with 10–100 μM Biot-(bAla)_4_-Pc 11 for 1 h at room temperature and washed twice in buffer B. Beads were then incubated with 100 ng recombinant CK2α in 100 µL of buffer A for 2 h at 4 °C . After three washes in 250 µL of buffer B, CK2 associated with Biot-(bAla)_4_-Pc 11 beads was evaluated using the CK2 kinase assay. Alternatively, Biot-(bAla)_4_-Pc 11 beads were incubated with extracts from WT or CK2β-depleted MCF-10A cells for 1 h at 4 °C. After 3 washes, beads were used in the CK2 kinase assay or analyzed by Western blot to detect the CK2 subunits.

### 3.6. In Vitro CK2α–CK2β Interaction Assay

The CK2α–CK2β interaction assay involved competition between plate-bound biotinylated MBP–CK2β and various cyclic peptides for binding to [^35^S] methionine-labelled CK2α. The assay was performed as previously described in [15].

### 3.7. Thermal Shift Denaturation Assay

CK2β-depleted MCF-10A cell lysates prepared in 50 mM Tris-HCl pH 7.5, 150 mM NaCl, and 2% glycerol supplemented with Complete Protease Inhibitor Cocktail (Sigma, #P8340) were divided into four aliquots with two being treated with 50 µM TAT or TAT-Pc 11 and the other two aliquots with CK2β or PBS (control). After 30 min incubation at room temperature, the respective lysates were divided into small (20 µL) aliquots in 0.2 mL microtubes and heated individually at different temperatures for 3 min (Thermocycler, Biometra) followed by cooling for 3 min in ice. WT MCF-10A cell lysates were heat-treated similarly. The heated lysates were centrifuged at 14,000× *g* for 20 min at 4 °C in order to separate the soluble fractions from precipitates. The supernatants were assayed for CK2 activity with the CK2β-independent peptide substrate.

### 3.8. Proximity Ligation Assay

In situ PLAs were performed using a Duolink kit (Olink Bioscience, Uppsala, Sweden) according to the manufacturer’s instructions with some modifications. MCF-10A cells were fixed in 4% paraformaldehyde for 10 min. The cells were then permeabilized with 0.1% Triton in Tris-buffered saline (TBS; 50 mM Tris, pH 7.6, 150 mM NaCl) and incubated with 100 mM glycine in phosphate-buffered saline (137 mM NaCl, 2.7 mM KCl, 10 mM Na_2_HPO_4_, and 1.8 mM KH_2_PO_4_, pH 7.4) for 20 min. Permeabilized cells were incubated overnight at 4 °C with primary antibodies diluted as follows: mouse CK2α 1:250 and rabbit CK2β 1:50. Cells were washed three times in TBS with 0.05% Tween-20 for 5 min each with gentle agitation. Secondary antibodies conjugated with oligonucleotides, PLA probe anti-mouse MINUS and PLA probe anti-rabbit PLUS, were added to the cells and incubated for 90 min at 37 °C in a humidity chamber. Finally, after ligation and amplification steps, cells were counterstained with the DNA-binding dye Hoechst and Phaloïdine-488 for actin staining (Molecular Probes, Thermo Fisher Scientific, Courtaboeuf, France). Images were observed using a Zeiss Apotome microscope and analyzed using a Zen Pro imaging software (Zeiss, Oberkochen, Germany). Quantification was performed using the BlobFinder software (V3.2, Swedish University of Agricultural Sciences, Uppsala University) [29]. Negative controls were one primary antibody with both of the secondary antibodies.

### 3.9. Cell Viability Assay

Cells were seeded in 96-well microtiter plates at a concentration of 1 × 10^5^ cells/mL and were allowed to attach for 24 h at 37 °C in 5% CO_2_. Cells were then exposed to different concentrations of TAT-Pc 13 for the indicated time. Cell viability was evaluated using the fluorescence-based LIVE&DEAD™ assay (Molecular Probes, Thermo Fisher Scientific, Courtaboeuf, France) according to the manufacturer. Images were taken with an Apotome-equipped Zeiss Axioimager microscope (Zeiss, Oberkochen, German) recording the fluorescence at 530 nm (Live cells) and 645 nm (dead cells) respectively using a FluoStar Optima plate reader (BMG LabTech, Ortenberg, Germany).

Alternatively, cell viability was measured using the PrestoBlue^®^ assay (Invitrogen, Carlsbad, CA, USA). The microtiter plates containing the treated cells were incubated for 1 h with 10 µL PrestoBlue. The fluorescence was recorded at 580 nm using a FluoStar Optima plate reader (BMG LabTech, Ortenberg, Germany).

## 4. Conclusions

The irreversible nature of the CK2 holoenzyme formation has been challenged by both its crystal structure [11] and live-cell imaging studies [12]. In addition, free populations of each CK2 subunit have been identified in several organs [30], and differential subcellular localizations have also been reported for CK2α and CK2β. Since the free catalytic subunit and the holoenzyme exhibit divergent substrate preferences, it could be predicted that such a balance is crucial to control the numerous cellular processes that are governed by this multifaceted enzyme [31]. The ability to interfere with specific protein–protein interactions has already provided powerful means of influencing the functions of selected proteins within the cell [32]. Consequently, it is expected that perturbating the CK2α/CK2β interface with artificial ligands might suppress specific CK2 holoenzyme functions providing a less toxic approach than total CK2 enzymatic inhibition. In a previous study, the presence within this interface of a small hydrophobic cavity on CK2α led us to a structure-based design of a CK2β-derived Pc peptide that can efficiently antagonize in vitro the high-affinity CK2 subunit interaction. To evaluate the potency and impact of the selective disruption of CK2α/CK2β interaction in a biologically relevant context, we describe here a cell-permeable version of Pc (TAT-Pc 13), exploring its molecular mechanisms of action and the perturbations of the signaling pathways that it induces in intact cells. Our study shows that TAT-Pc 13 rapidly accumulates into living cells, promoting the disruption of the CK2 subunit interaction, thereby antagonizing specific functions of CK2β. Intriguingly, cell treatment with TAT-Pc 13 rapidly induces dramatic cellular perturbations leading to caspase-independent cell death. In particular, we observed that TAT-Pc 13 induced a phosphorylation on Thr145 of the p21 protein, associated with its nuclear accumulation. It has been reported that p21 is regulated by phosphorylation and several protein kinases such as AKT [33], Pim1 [34] or the death-associated protein kinase DAPK3 [35,36] have been shown to stabilize p21 through phosphorylation of Thr145. A functional relationship between CK2 and the Pim1 kinase has not been reported yet. Moreover, since we could not detect any AKT activation, this kinase is probably not implicated in TAT-Pc 13-induced p21 phosphorylation. DAPK3, also called ZIP kinase (ZIPK), has been implicated in interferon γ-and TNFα/Fas-induced cell death [37] and low ZIPK expression was correlated with increased migration and invasion in breast cancer cells [38]. Interestingly, a potential link between CK2 and the ZIPK has been established. The transcription and splicing factor CDC5 was shown to be an interacting partner of ZIPK and both proteins co-localize with CK2 in speckle-like structures. Moreover, CDC5 is associated with and efficiently phosphorylated by CK2 suggesting a potential CK2-CDC5-ZIPK-p21 axis in triggering cell death [39]. Nevertheless, further investigations in this direction will be beneficial in understanding the implication of p21 in the specific cellular mechanisms leading to cell death upon TAT-Pc 13 treatment. Unexpectedly, in contrast to the cytotoxic effect of CX-4945, TAT-Pc 13-induced cell death did not require caspase activation. Therefore, future studies will focus on defining the cell death modality activated following disruption of the CK2 subunit interaction. As a preliminary clue, the results illustrated in Supplementary Materials Figure S2 show that TAT-Pc 13-induced cell death was prevented by Necrostatin-1, a selective small molecule inhibitor of necroptosis [40].

In summary, TAT-Pc 13 can significantly facilitate functional studies, in particular to identify CK2β-targeted CK2 substrates in living cells using phosphoproteomic approaches. Selective disruption of CK2α/CK2β interaction could also find important applications to pharmacologically test the importance of this interaction in normal and tumor cell biology. With the help of structure-based rational design, TAT-Pc 13 may also serve as a lead for the rational design of function-specific drugs that disrupt some actions of CK2. It is expected that such compounds will be substrate selective, inhibiting through an unconventional way the activity of the kinase against a subset of its substrates but leaving others intact.

## Supplementary Materials

The following are available online at http://www.mdpi.com/1424-8247/10/1/16/s1, Figure S1: Cellular effects of TAT-Pc 13, Figure S2: Inhibition of TAT-Pc-induced cell death.
