# Supplementary material for: In Search of Small Molecule Inhibitors Targeting the Flexible CK2 Subunit Interface
Source: Pharmaceuticals (Basel). 2017 Feb 3;10(1):16. doi: 10.3390/ph10010016 (PMC5374420; doi:10.3390/ph10010016)
Supplement: Supplementary file 1 [file pharmaceuticals-10-00016-s001.pdf]

Supplementary Materials: In Search of Small Molecule Inhibitors Targeting the Flexible CK2 Subunit Interface

Benoit Bestgen, Zakia Belaid-Choucair, Thierry Lomberget, Marc Le Borgne, Odile Filhol and Claude Cochet

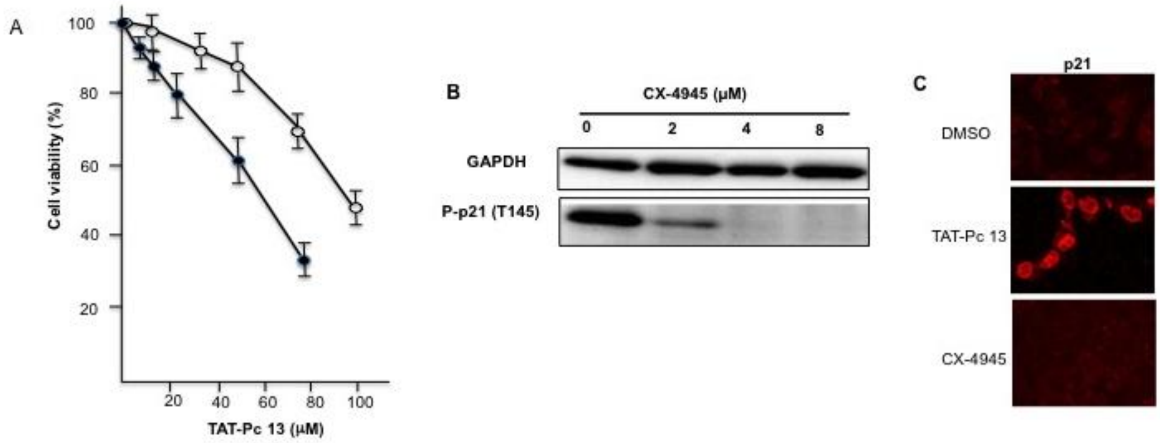

Figure S1. Cellular effects of TAT-Pc 13. (**A**) MDA MB-231 cells (•-•) or HeLa cells (○-○) were incubated for 6 h with increasing concentrations of TAT-Pc 13 and cell death was measured using the PrestoBlue assay as described in Material and Methods; (**B**) 786-O cells were treated for 12 h with increasing concentrations of CX-4945, lysed and analyzed by Western blot; (**C**) 786-O cells were treated with 20 µM TAT-Pc 13 or 5 µM CX-4945 and immunostained for p21 expression.

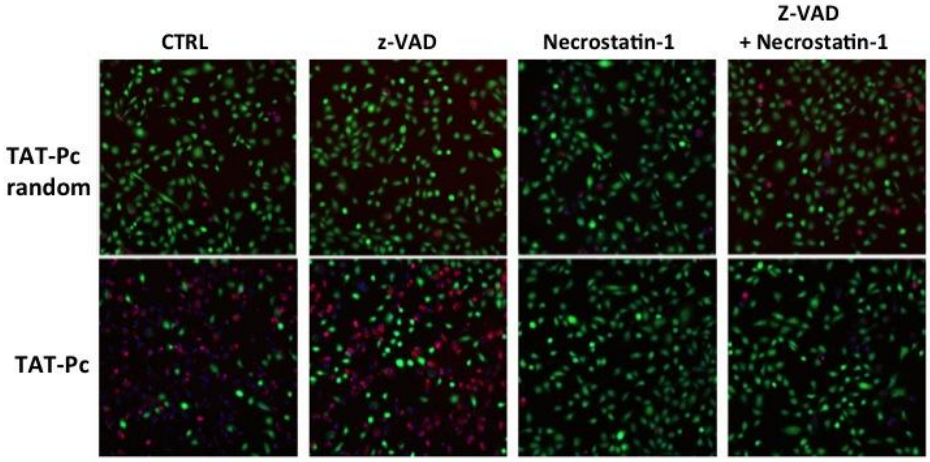

Figure 2. Inhibition of TAT-Pc-induced cell death. MCF-10A cells were pre-treated in the absence (CTRL) or presence of 20 µM z-VAD or 30 µM Necrostatin-1 or 20 µM z-VAD + 30 µM Necrostatin-1 for 5 h and then incubated for 4 h with 25 µM random TAT-Pc 13 or TAT-Pc 13 and cell viability was evaluated as in Figure 8A.
